# Automated segmentation of multifocal basal ganglia T2*-weighted MRI hypointensities

**DOI:** 10.1016/j.neuroimage.2014.10.001

**Published:** 2015-01-15

**Authors:** Andreas Glatz, Mark E. Bastin, Alexander J. Kiker, Ian J. Deary, Joanna M. Wardlaw, Maria C. Valdés Hernández

**Affiliations:** a Brain Research Imaging Centre, Neuroimaging Sciences, University of Edinburgh, Western General Hospital, Crewe Road, Edinburgh EH4 2XU, UK; b SINAPSE Collaboration, Brain Research Imaging Centre, Neuroimaging Sciences, University of Edinburgh, Western General Hospital, Crewe Road, Edinburgh EH4 2XU, UK; c Centre for Clinical Brain Sciences, University of Edinburgh, Edinburgh, UK; d Centre for Cognitive Ageing and Cognitive Epidemiology, University of Edinburgh, Edinburgh EH8 9JZ, UK; e Department of Psychology, University of Edinburgh, Edinburgh EH8 9JZ, UK

**Keywords:** Magnetic resonance imaging (MRI), Basal ganglia, Mineralization, Ageing, Focal T2*-weighted MRI hypointensities, Automated segmentation, Outlier detection

## Abstract

Multifocal basal ganglia T2*-weighted (T2*w) hypointensities, which are believed to arise mainly from vascular mineralization, were recently proposed as a novel MRI biomarker for small vessel disease and ageing. These T2*w hypointensities are typically segmented semi-automatically, which is time consuming, associated with a high intra-rater variability and low inter-rater agreement. To address these limitations, we developed a fully automated, unsupervised segmentation method for basal ganglia T2*w hypointensities. This method requires conventional, co-registered T2*w and T1-weighted (T1w) volumes, as well as region-of-interest (ROI) masks for the basal ganglia and adjacent internal capsule generated automatically from T1w MRI. The basal ganglia T2*w hypointensities were then segmented with thresholds derived with an adaptive outlier detection method from respective bivariate T2*w/T1w intensity distributions in each ROI. Artefacts were reduced by filtering connected components in the initial masks based on their standardised T2*w intensity variance. The segmentation method was validated using a custom-built phantom containing mineral deposit models, i.e. gel beads doped with 3 different contrast agents in 7 different concentrations, as well as with MRI data from 98 community-dwelling older subjects in their seventies with a wide range of basal ganglia T2*w hypointensities. The method produced basal ganglia T2*w hypointensity masks that were in substantial volumetric and spatial agreement with those generated by an experienced rater (Jaccard index = 0.62 ± 0.40). These promising results suggest that this method may have use in automatic segmentation of basal ganglia T2*w hypointensities in studies of small vessel disease and ageing.

## Highlights

• A novel method segmented focal T2*-weighted MRI hypointensities automatically.
• The method was validated with MRI of a novel phantom and 98 elderly subjects.
• The subject masks from the method and an experienced rater overlapped substantially.
• The method is potentially useful for research into small vessel disease and ageing.

## Introduction

Focal hypointensities appear as a frequent feature on T2*-weighted (T2*w) MRI in the basal ganglia of elderly, otherwise healthy, subjects (Glatz et al., 2013). These features are believed to arise from mineralisation in and around penetrating arteries and perivascular spaces (Casanova and Araque, 2003; Morris et al., 1992; Slager and Wagner, 1956), which are possibly of ischemic origin (Janaway et al., 2014). Harder et al. (2008), who studied focal basal ganglia hypointensities on susceptibility-weighted imaging (SWI), found that their degree and hypointensity increase with age, while Penke et al. (2012) demonstrated that their volume correlated negatively with cognitive ability in both youth and older age in a group of 143 community-dwelling subjects in their seventies. Other studies, such as Aquino et al. (2009) and Li et al. (2014), that have investigated the appearance of the basal ganglia in non-demented elderly subjects on gradient-echo MRI have found that this structure becomes more hypointense with age due to iron storage (Hallgren and Sourander, 1958). However, van Es et al. (2008) reported that increased basal ganglia iron might also be associated with other age-related changes in the brain, such as white matter T2-weighted (T2w) hyperintensities.

MRI has become the de facto standard for assessing iron and mineral deposits in vivo (Haacke et al., 2005; Schenck and Zimmerman, 2004; Valdés Hernández et al., 2012). These trace metal deposits accelerate the realignment of water proton spins in the direction of the main magnetic field and their dephasing in the transverse plane. This causes a localized shortening of T1, T2, and T2* relaxation times and can lead to focal hyperintensities on T1-weighted (T1w) volumes, and focal hypointensities on T2w and T2*w volumes. However, trace metal deposits, such as ferritin, that are separated from water protons by a water-soluble shell predominantly affect the contrast of T2w and T2*w MRI, whereas they appear isointense on T1w MRI (inner and outer sphere theory; Brass et al., 2006; Schenck, 2003). The T2w and T2*w contrast of trace metal deposits depends on their magnetic susceptibility and their particle radius relative to the average water proton diffusion path length (Weisskoff et al., 1994).

Focal basal ganglia T2*w hypointensities appear predominantly iso- to slightly hypointense on T1w MRI and isointense on T2w MRI which indicates that the underlying mineral deposits are more water-insoluble than ferritin (Vymazal et al., 2000), and consist of aggregated trace metals since this increases reversible dephasing of diffusing water protons (Sedlacik et al., 2014; Weisskoff et al., 1994). Subregions of basal ganglia T2*w hypointensities can also appear very hypointense on T1w MRI which has been linked to advanced mineralization of the underlying tissue, such as calcification (Henkelman et al., 1991; Slager and Wagner, 1956; Valdés Hernandéz et al., 2014).

Methods for analysing basal ganglia T2*w hypointensities either determine the hypointensity of the whole basal ganglia (Jasinschi et al., 2006; Parsey and Krishnan, 1997; van Es et al., 2008) or the volume of focal T2*w hypointensities in individual structures (Valdés Hernández et al., 2011). The former method first classifies all voxels as hypointense that fall below a T2*w threshold which is either derived from the T2*w signal intensities of the red nucleus or globus pallidus. The ratio of hypointense to basal ganglia structure voxels quantifies the degree of hypointensity of each structure. The latest improvements in this method produce reliable results that are in good agreement with those generated by experienced raters (Jasinschi et al., 2006). On the other hand, focal T2*w hypointensities in the basal ganglia are still typically segmented semi-automatically (Valdés Hernández et al., 2011). An experienced rater first refines a T2*w threshold equal to the median T2*w signal intensity of the globus pallidus to exclude most artefacts. The rater then manually removes the remaining artefacts based on the visual appearance of focal T2*w hypointensities on T2*w and T1w MRI. An alternative method has also been developed that produces colour maps of the brain with minimum variance quantization of co-registered T2*w and fluid attenuated inversion recovery (FLAIR) volumes (Valdés Hernández et al., 2010). Haemosiderin deposits, which appear green on these maps, are manually identified and included in the final masks. However, validation of these methods shows that both are very time-consuming and associated with high intra-rater variability and low inter-rater agreement (Valdés Hernández et al., 2011).

In this study we therefore developed a fully automated method for segmenting basal ganglia T2*w hypointensities to address the limitations of the previously developed semi-automatic methods. We then investigated the effect of method parameters on the segmentation results in a custom-designed phantom employing several mineral deposit models. The method was also validated with MRI data from a group of community-dwelling subjects in their seventies with a wide range of basal ganglia T2*w hypointensities which have been manually and semi-automatically segmented by two experienced raters. The masks from the manual segmentation were then used to optimise the parameters of the fully automated method, and to assess and compare the accuracy and precision of the masks from the fully automated and semi-automated segmentation.

## Methods

**Fig. 1:**
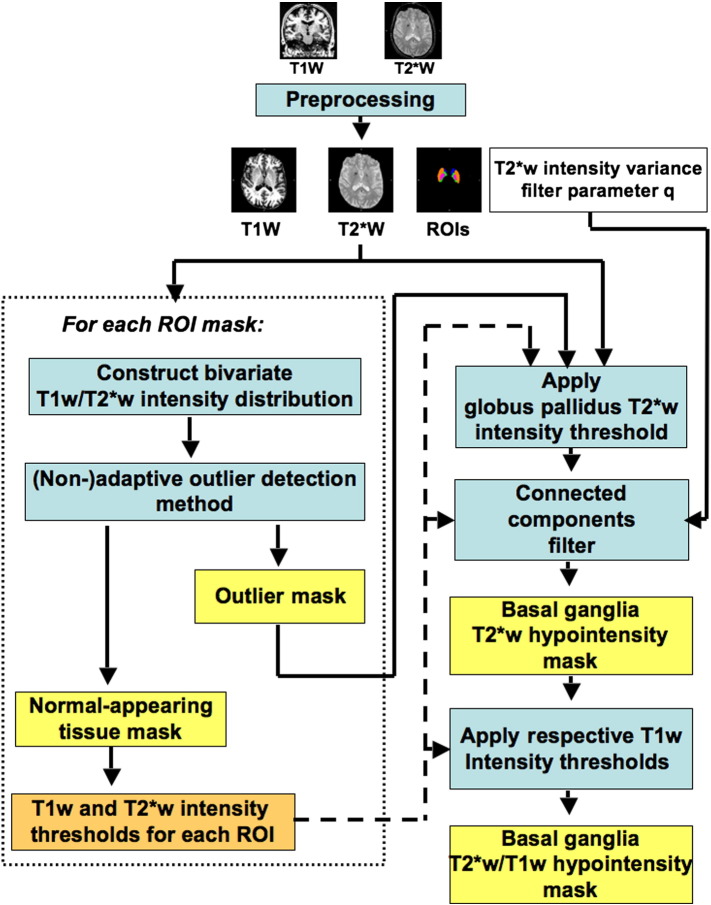
Overview of the fully automated method for segmenting basal ganglia T2*w hypointensities. The method requires structural T2*w and T1w volumes as input. The preprocessing step removes non-brain structures, reduces non-anatomical T2*w and T1w intensity variations (bias field) and automatically generates ROIs for the basal ganglia and adjacent internal capsule. Then T2*w and T1w thresholds are automatically derived with an unsupervised outlier detection method from the T2*w and T1w intensity distribution of each ROI. Initial T2*w hypointensity masks are obtained by applying the T2*w threshold to the T2*w volume. The connected components of these masks are identified and filtered according to their T2*w intensity variance, which yields the final T2*w hypointensity masks. Additionally, subregions of basal ganglia T2*w hypointensities that appear hypointense on T1w MRI are segmented since these possibly indicate advanced mineralization, such as calcification.

Fig. 1 shows an overview of the fully automated method for segmenting basal ganglia T2*w hypointensities. This method generates basal ganglia T2*w hypointensity masks, which possibly indicate basal ganglia mineral deposits (Penke et al., 2012), as well as T2*w/T1w hypointensity masks, which possibly indicate regions of advanced mineralisation, such as calcification (Valdés Hernandéz et al., 2014). The method generates masks in three steps. Firstly, the structural T2*w and T1w input volumes are preprocessed, which produces co-registered T2*w and T1w volumes, as well as regions-of-interest (ROI) masks. Secondly, T2*w and T1w thresholds are derived for segmenting focal T2*w hypointensities. Lastly, initial output masks are created by applying these thresholds to the co-registered T2*w and T1w volumes, which are subsequently filtered to reduce thresholding artefacts.

The preprocessing pipeline was mainly implemented in GNU Bash (www.gnu.org) with tools from FSL 5.0 (www.fmrib.ox.ac.uk/fsl) and N4 (www.itk.org), whereas the main processing pipeline was implemented in Matlab 2011b (Natick, MA, USA) with the LIBRA (Verboven and Hubert, 2005) and NIFTI tools (Matlab Central, File ID: #8797). The developed software is available at github.com/aglatz.

### Preprocessing pipeline for structural T2*w and T1w MRI

A previously published preprocessing pipeline (Glatz et al., 2013) was used to obtain co-registered T1w and T2*w volumes, as well as caudate, putamen, globus pallidus and adjacent internal capsule masks, which were combined in a ROI mask set. In short, non-brain structures visible on T2*w volumes were automatically removed with FSL BET (Smith, 2002). Non-brain structures visible on T1w volumes were removed by transforming the brain masks created by FSL BET from T2*w to T1w space and by applying them to the corresponding T1w volumes. N4 was used for bias-field correction of all volumes and the T1w volumes were affine registered to the corresponding T2*w volumes using FSL FLIRT (Jenkinson et al., 2002).

To generate the ROI mask set, the basal ganglia nuclei and the thalamus were automatically segmented on the original T1w volumes using FSL FIRST (Patenaude et al., 2011). All structural masks were then linearly transformed from T1w to T2*w space with FSL FLIRT and the previously obtained transformation matrices. Additional internal capsule masks were created by dilating the globus pallidus masks towards the centre of the brain with half disk shaped structural elements of 6 mm radius and then removing regions of these masks that intersected with the union of caudate, putamen, thalamus and globus pallidus masks. The final ROI mask set consisted of four ROI masks, *M*_*l*_^ROI^ ⊂ *M*, with the structure labels *l* ∈ {cn, pu, gp, ic} corresponding to the caudate nucleus (cn), putamen (pu), globus pallidus (gp) and adjacent internal capsule (ic), where *M* ⊂ **Z**^3^ indexes the MRI volume voxel lattice.

### Automated threshold selection for segmenting focal T2*w hypointensities

The T2*w intensities of tissue *l* with focal T2*w hypointensities can be modelled as

(1) $$G_{l}=\left(1-\xi \right)G_{l}^{\mathrm{norm}}+\xi G_{l}^{\mathrm{hypo}}\text{,}$$

where the cumulative distribution function of the T2*w intensities *G*_*l*_ are a mixture of normal appearing T2*w tissue intensities and T2*w hypointensities with the cumulative distribution functions *G*_*l*_^*norm*^ and *G*_*l*_^*hypo*^, and 0 ≤ *ξ* ≤ 1 as the mixture weight. If both mixture components are normally distributed then methods, such as mixture discriminant analysis (Fraley and Raftery, 2002), can derive an optimal T2*w threshold for dividing the T2*w tissue intensities into normal appearing T2*w tissue intensities and T2*w hypointensities. However, as previously noted (Glatz et al., 2013), T2*w hypointensity distributions typically do not resemble normal distributions, their shapes are variable and their mixture weights are very small (*ξ* < < 1), hence identifyingtheir underlying parametric distributions is challenging. Therefore T2*w hypointensities were considered outliers of the normal appearing T2*w tissue intensity distribution, which is approximately normally distributed in cases where the signal-to-noise ratio (SNR) is larger than 2 (Gudbjartsson and Patz, 1995).

In this study a previously published robust multivariate outlier detection method (Filzmoser et al., 2005) was adapted for co-registered T2*w and T1w MRI data as described in detail in Appendix A. This method derives the T2*w and T1w thresholds

(2) $$\begin{array}{l} s_{l,T2*w}^{\mathrm{thresh}}=s_{l,T2*w}^{\mu }-s_{l,T2*w}^{\sigma }{\overset{⌣}{d}}_{l}^{\mathrm{crit},m}\\ s_{l,T1w}^{\mathrm{thresh}}=s_{l,T1w}^{\mu }-s_{l,T1w}^{\sigma }{\overset{⌣}{d}}_{l}^{\mathrm{crit},m} \end{array}$$

for segmenting focal T2*w hypointensities and their subregions that appear hypointense on T1w MRI. The robust means *s*_*l*,T2 ⁎ w_^*μ*^, *s*_*l*,T1w_^*μ*^ and standard deviations *s*_*l*,T2 ⁎ w_^*σ*^, *s*_*l*,T1w_^*σ*^ of the normal appearing T2*w and T1w tissue intensity distributions were estimated with the minimum covariance determinant (MCD) method (Rousseeuw, 1999). The z-score of the critical distance ${\overset{⌣}{d}}_{l}^{\mathrm{crit},m}$ was constant or variable in case of the non-adaptive or adaptive version of the outlier detection method *m* ∈ {*na*, *ad*}. A refinement of the constant z-score accounted for the finite sample size of the normal appearing T2*w and T1w tissue intensity distributions.

The contrast-to-noise ratio (CNR) corresponding to the thresholds in Eq. (2) is

(3) $$CNR_{l,t}^{\mathrm{thresh},m}=\frac{\left|s_{l,t}^{\mathrm{thresh},m}-s_{l,t}^{\mu }\right|}{s_{l,t}^{\mathrm{noise}}}=\frac{\left|s_{l,t}^{\sigma }\right|}{s_{l,t}^{\mathrm{noise}}}{\overset{⌣}{d}}_{l}^{\mathrm{crit},m}=CNR_{l,t}^{\sigma }{\overset{⌣}{d}}_{l}^{\mathrm{crit},m}$$

with *t* ∈ {T2 * w, T1w}. It depends on the CNR corresponding to the spread of the normal appearing T2*w and T1w tissue distributions as well as the z-score of the critical distance, hence it depends on the MRI sequence parameters influencing the image noise, the estimation method of the spread of the normal appearing T2*w and T1w tissue distributions, as well as the sample size of this distribution in the case of the adaptive outlier detection method. However, the CNR corresponding to the thresholds is independent of the mean T2*w and T1w tissue intensities, which both are known to decrease with age due to age-related tissue changes, such as iron accumulation (Aquino et al., 2009).

### Segmentation and filtering of focal T2*w hypointensities

The T2*w and T1w thresholds (Eq. (2)) were derived individually for each ROI. Similar to van Es et al. (2008) and Valdés Hernández et al. (2011), the T2*w hypointensities of all ROIs were then segmented with the respective T2*w threshold of the globus pallidus since it represents the lowest and hence most conservative T2*w threshold, which then created initial T2*w hypointensity masks. The thresholding artefacts of these masks were then eliminated with a connected components filter, which yielded the final T2*w hypointensity masks. This filter is based on the observation that a rater corrects the initial T2*w hypointensity masks by selectively removing individual focal T2*w hypointensities, i.e. connected components of the initial T2*w hypointensity masks. One of the criteria the rater used to judge whether a focal T2*w hypointensity of the initial mask needed to be removed was the smoothness of its appearance on T2*w MRI. Preliminary investigations have shown that the rater preferably removed focal T2*w hypointensities from the initial masks if they appeared too smooth, i.e. if the T2*w hypointensities were too similar. Therefore the connected components filter first identified the connected components of the initial masks and then removed connected components whose standardised T2*w intensity variance

(4) $$q_{l,h}={\left(\frac{s_{h,T2*w}^{\mathrm{hypo},\sigma }}{s_{l,T2*w}^{\mathrm{norm},\sigma^{loc}}}\right)}^{2}$$

was below a threshold *q*. Here $s_{h,T2*w}^{\mathrm{hypo},\sigma }$ is the standard deviation of the T2*w hypointensities of the connected component *h* and $s_{l,T2*w}^{\mathrm{norm},\sigma^{loc}}$ is the local standard deviation of the normal appearing T2*w tissue intensities of the structure where the connected component is located. A threshold *q* > 0 also implicitly removes connected components that have a size of a single voxel since the standardised T2*w intensity variance of such connected components is 0.

The final T2*w hypointensity masks and T2*w/T1w hypointensity masks generated in the segmentation and filtering step are furthermore denoted *M*_*l*,T2 ⁎ w_^*hypo*^ and *M*_*l*,T1w_^*hypo*^. The latter select the subregions of T2*w hypointensities that appear hypointense on T1w MRI, hence *M*_*l*,T1w_^*hypo*^ ⊂ *M*_*l*,T2 ⁎ w_^*hypo*^. These masks were created with the T1w thresholds (Eq. (2)) corresponding to the structures, where the individual T2*w hypointensities were located. The segmentation and filtering step is described in further detail in Appendix B.

## Validation

The presented automated method for segmenting focal T2*w hypointensities was validated with co-registered T1w and T2*w volumes acquired from (i) a custom-designed MRI phantom containing doped gel beads as models for basal ganglia mineralizations and (ii) 98 community-dwelling subjects in their seventies recruited from the Lothian Birth Cohort 1936 (LBC1936; Deary et al., 2007). In both cases the multifocal T2*w hypointensities were automatically segmented with the presented method. The automatically generated masks were compared with reference masks, which in the case of the subjects, were produced by an experienced rater. The purpose of the phantom was to analyse the appearance of the basal ganglia mineralization models on T2*w MRI, as well as to investigate the effect of the adaptive outlier detection method and the connected components filter on the segmentation results. The validation of the software with subject data was carried out to identify the optimal connected components filter parameters for obtaining basal ganglia T2*w hypointensity masks that best resemble those from the rater, as well as to determine the accuracy and precision of the developed segmentation method.

### Validation with a custom-built phantom

#### Phantom design

**Fig. 2:**
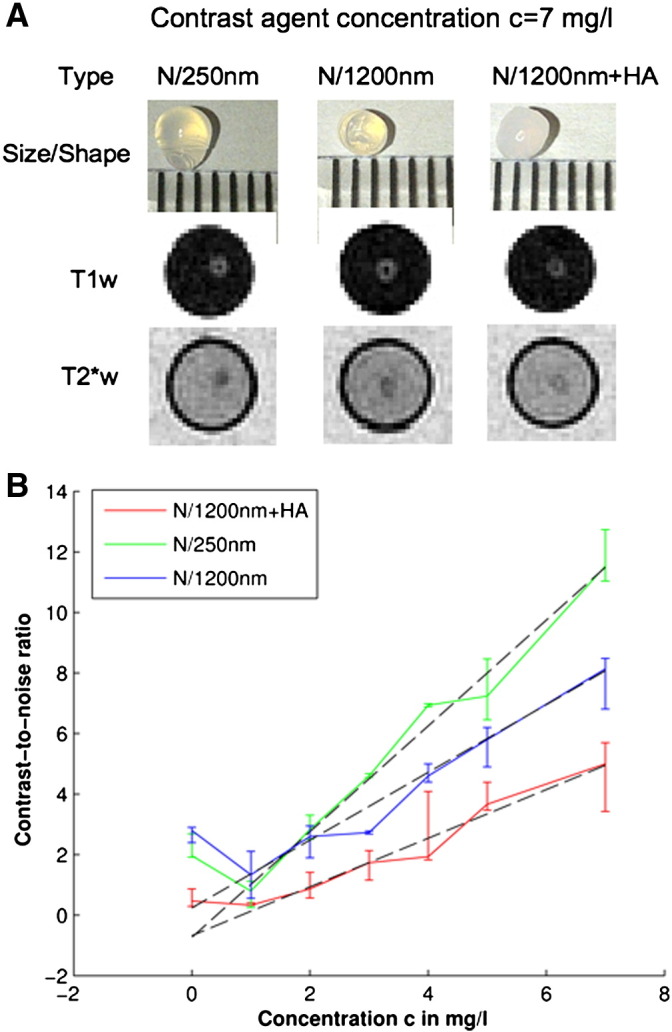
Phantom models of basal ganglia T2*w hypointensities and their appearance on T2*w MRI. Doped calcium alginate (CaAlg) gel beads were used as models for focal mineralizations in the basal ganglia (A). These gel beads were on average spherical with a diameter of 3 mm and either doped with the MRI contrast agent Nanomag-D 250 nm (N/250 nm), Nanomag-D 1200 nm (N/1200 nm), or Nanomag-D 1200 nm and hydroxyapatite nanocrystals (N/1200 nm + HA). These beads then appeared as focal hypo- and hyperintensities on T2*w and T1w MRI. The plot below (B) shows the median T2*w contrast-to-noise ratios (CNRs) of all three gel bead replicates (Eq. (5)) over the contrast agent concentrations, where the error bars indicate the respective interquartile ranges. The CNR increase with the contrast agent concentration was modelled with robust linear regression lines (dashed).

Three types of calcium alginate (CaAlg) gel beads (Fig. 2A) containing the MRI contrast agents Nanomag-D 250 nm (N/250 nm), Nanomag-D 1200 nm (N/1200 nm) and Nanomag-D 1200 nm mixed with hydroxyapatite (HA) nanocrystals (N/1200 nm + HA) were produced as models for mineral deposits in the basal ganglia. Seven gel beads of the same type containing varying amounts of Nanomag-D 250 nm or Nanomag-D 1200 nm (Table 1) were suspended in 1.8% w/v agarose in a subcompartment of the phantom. The final phantom consisted of 9 such subcompartments, which were sealed 10 ml BD syringes (www.medisave.co.uk), so that each gel bead type with the same contrast agent concentration was replicated three times. To provide head coil loading and to reduce susceptibility artefacts, these subcompartments were placed in 1.25 g/l CuSO_4_, 3.6 g/l NaCl solution and oriented parallel to the main magnetic field of the scanner.

Nanomag-D 250 nm (micromod Partikeltechnologie, Rostock, Germany) is a MRI contrast agent, which consists of 5–15 nm iron particles inside a dextran matrix with a diameter of 250 nm. Nanomag-D 1200 nm particles are produced by carefully aggregating Nanomag-D 250 nm with additional dextran as glue and have previously been used as a model of basal ganglia iron deposits (Sedlacik et al., 2014). The protocol for producing the CaAlg gel beads was based on that described by (Xie et al., 2010). In short, 2% (w/v) sodium alginate solutions were mixed either with N/250 nm, N/1200 nm, or N/1200 nm and a hydroxyapatite precursor, as summarized in Table 1, and were dripped with 10 ml BD syringes into 500 mM calcium chloride solutions. The droplets were transitioned in these solutions into approximately spherical gel beads with a mean diameter of 3 mm containing iron and hydroxyapatite nanocrystals as shown in Fig. 2A.

#### MRI protocol

The phantom was scanned on a GE Signa HDxt 1.5 T clinical scanner (General Electric, Milwaukee, WI, USA) using a self-shielding gradient set with maximum gradient of 33 mT/m and an eight-channel phased-array receive/transmit head coil. Table 2 shows the MRI protocol for the phantom, which consisted of T2*w (GRASS) and T1w (IR-prep SPGR) sequences. This protocol was the same as that used to image the subjects except that the field-of-view was smaller and the slices were thicker to reduce Gibbs ringing artefacts caused by the subcompartment walls of the phantom. T2*w and T1w sequences were prescribed to image exactly the same volume containing all the gel beads, which produced naturally co-registered T2*w and T1w volumes. The typical appearance of the gel beads on T2*w and T1w MRI is shown in Fig. 2A. Unlike basal ganglia T2*w hypointensities (Glatz et al., 2013), all CaAlg gel beads were clearly visible on T1w MRI as focal hyperintensities, which had no impact on their automated segmentation and was exploited for creating the reference masks as described further below.

#### Image preprocessing and semi-automatic ROI segmentation

N4 was used for the bias field correction of the T2*w and T1w volumes (Tustison et al., 2010). The T1w volume was then resampled using FSL FLIRT with sinc interpolation (Jenkinson et al., 2002) to match the resolution of the T2*w volume. ROI masks were semi-automatically created with the T1w volume as described below and then resampled using FSL FLIRT with nearest neighbour interpolation (Jenkinson et al., 2002).

Initial ROI masks were produced by thresholding the bias-field corrected T1w volume with a threshold equal to 80% of the mean T1w signal intensity of the CuSO_4_/NaCl solution. The obtained mask was first dilated using a spherical kernel with 6 mm diameter to close holes caused by the T1w hyperintensities of the gel beads and then eroded using a spherical kernel with 12 mm diameter to remove artefacts from the plastic walls of the subcompartments. The final ROI masks were obtained after manually removing remaining artefacts and assigning unique labels to the individual masks of each subcompartment.

#### Segmentation and quality control of the CaAlg gel beads on T2*w MRI

The CaAlg gel bead reference mask was obtained by automatically placing spherical shaped masks with the average diameter of the gel beads at the locations of the T1w hypointensities created by the gel beads. For the quality control of the CaAlg gel beads this mask was subsequently used to calculate the CNRs on T2*w MRI of all gel beads, which should increase approximately linearly per gel bead type with contrast agent concentration due to the short echo time and the low contrast agent concentrations.

The initial locations of these T1w hypointensities were estimated from masks which were created by logically inverting the initial ROI masks and combining them with the final ROI masks with a logical AND operation. The connected components of the resulting masks then corresponded to most of the T1w hypointensities caused by the CaAlg gel beads. Missing connected components or connected components associated with artefacts were added or removed manually. Subsequently, the connected components were replaced by the spherical masks with a diameter of 3 mm, which were typically smaller than the hyperintensities visible on the original T1w volumes due to partial volume effects (Rexilius and Peitgen, 2008). Finally, the spherical masks *M*_*v*,*w*,*l*,T2 ⁎ w_^*hypo*,*ref*^, where *v* ∈ {1, 2, …, 7} is the gel bead index, *w* ∈ {1, 2, 3} is the replicate index, and *l* ∈ {1, 2, 3} is the ROI index, were centred on the corresponding T1w hypointensities. The optimal position of a spherical mask was estimated by translating the masks along all coordinate system axes to find the position where the mean T1w intensities of all voxels selected by this mask became a maximum.

The mean CNR of all CaAlg gel bead replicates on T2*w MRI then calculated with

(5) $${CNR}_{v,l,T2*w}=\left(\frac{1}{3}\sum_{w=1}^{3}\left|s_{v,w,l,T2*w}^{\mathrm{hypo},\mu }-s_{w,l,E2*w}^{\mathrm{norm},\mu }\right|\right)/s_{T2*w}^{\mathrm{noise}}\text{,}$$

where *s*_*v*,*w*,*l*,T2 ⁎ w_^*hypo*,*μ*^ is the robust mean T2*w intensity of an individual CaAlg gel bead selected by the masks *M*_*v*,*w*,*l*,T2 ⁎ w_^*hypo*,*ref*^, *s*_*w*,*l*,T2 ⁎ w_^*norm*,*μ*^ is the robust mean intensity of the agarose in the respective subcompartment, and *s*_T2 ⁎ w_^*noise*^ is the T2*w image noise (Firbank et al., 1999). The increase of the mean CNR per gel bead type with the contrast agent concentration was estimated with robust linear regression lines (Matlab function ‘robustfit()’).

#### Automated segmentation of focal T2*w hypointensities in the phantom

Focal T2*w hypointensities were segmented in each subcompartment of the phantom using the original and bias-field corrected T2*w and T1w volumes with the respective T2*w thresholds from the non-adaptive and adaptive version of the outlier detection method (Automated threshold selection for segmenting focal T2⁎w hypointensities section). To investigate the effect of the connected components filter on the segmentation results, the initial masks were also filtered with the connected components filter parameters *q* = 0, 0.1,…, 1.5. To aid further analysis, the connected components (six-connected neighbourhood) of the generated masks *M*_*w*,*l*,T2 ⁎ w_^*hypo*^(*b*, *m*, *q*), where *b* ∈ {*orig*, *bfc*} created with the original or bias-field corrected T2*w and T1w volumes were also identified and labelled with the Matlab function ‘bwlabeln()’.

#### Comparison of the non-adaptive and adaptive outlier detection methods

For the comparison of the non-adaptive and adaptive version of the outlier detection method the agreement between the CaAlg gel bead reference mask and the corresponding T2*w hypointensity masks, which were generated on the original and bias-field corrected volumes, were assessed. The number of segmented T2*w hypointensities that corresponded to gel beads, as well as those that represented thresholding artefacts were counted. A connected component of the generated masks was considered to be associated with a gel bead if its mask overlapped at least 50% with the corresponding reference mask, i.e. the regional sensitivity was greater than 0.5 (Shattuck et al., 2009). The spatial agreement between the CaAlg gel bead reference and generated masks was quantified with the Jaccard index (Shattuck et al., 2009)

(6) $$J_{l}\left(b,m,q\right)=\frac{\left|M_{l,T2*w}^{\mathrm{hypo}}\left(b,m,q\right)\cap M_{l,T2*w}^{\mathrm{hypo},ref}\right|}{\left|M_{l,T2*w}^{\mathrm{hypo}}\left(b,m,q\right)\cup M_{l,T2*w}^{\mathrm{hypo},ref}\right|}\text{,}$$

which is 0 in situations where these masks are completely disjointed or 1 if they are identical. Lastly, the minimum CNRs of the segmented CaAlg gel beads and the CNR of the respective T2*w thresholds (Eq. (3)) were calculated to investigate their agreement.

#### Analysis of the blooming artefacts around the CaAlg gel beads

To analyse how the blooming artefacts depend on the gel bead type and MRI contrast agent concentration, the apparent volume increase of the doped CaAlg gel beads on T2*w MRI was calculated and plotted over the MRI contrast agent concentration.

Firstly, the mean volumes of the connected components masks *M*_*h*,*w*,*l*,T2 ⁎ w_^*hypo*^(*b* = *bfc*, *m*, *q* = 0) associated with gel beads containing the same amount and type of contrast agent were

(7) $$V_{h,l}\left(m\right)=\frac{1}{3}\sum_{w=1}^{3}\left|M_{h,w,l,T2*w}^{\mathrm{hypo}}\left(b=bfc,m,q=0\right)\right|V^{vox}$$

with *V*^*vox*^ as the voxel volume. As the connected components were on average spherical their diameter *D*_*h*,*l*_(*m*) could be estimated with the volume formula of a sphere. The apparent volume increase of the gel beads on T2*w MRI was then quantified with the relative gel bead diameter *D*_*h*,*l*_^*rel*^(*m*) = *D*_*h*,*l*_(*m*)/*D*^*bead*^ with *D*^*bead*^ ≈ 3 mm, which was then plotted over the contrast agent concentration.

#### Analysis of the connected components filter characteristics

The characteristic filter functions of the connected components filter indicate the change in the spatial agreement between the masks *M*_*l*,T2 ⁎ w_^*hypo*^(*b* = *bfc*, *m* = *ad*, *q*) and the CaAlg gel bead reference mask with the filter parameter *q*. To construct the characteristic filter functions of each ROI, the spatial agreement between each generated mask and the reference mask was quantified with the standardised Jaccard index

(8) $$J_{l}^{std}\left(q\right)=\frac{J_{l}\left(q\right)}{J_{l}\left(q=0\right)}\text{,}$$

with *J*_*l*_(*q*) := *J*_*l*_(*b* = *bfc*, *m* = *ad*, *q*) from Eq. (6). The relative Jaccard index was evaluated at *q* = 0, 0.1, …, 1.5 and then plotted over the parameter *q*. The FWHM of a characteristic filter function was the parameter *q*_*l*_^*FWHM*^, where *J*_*l*_^*std*^(*q*_*l*_^*FWHM*^) = 0.5.

### Validation with subject data

#### Subject cohort and validation sample

The LBC1936 and associated MRI protocol (partly included in Table 2) are described in detail in Deary et al. (2007, 2012) and Wardlaw et al. (2011). In short, the LBC1936 is a longitudinal study of cognitive ageing that originally recruited a group of 1091 community-dwelling individuals resident in the Edinburgh and Lothian areas of Scotland who were born in 1936. Seven hundred of them were scanned at a mean age of 72.5 years (SD = 0.7 years) with a published protocol on a GE Signa HDxt 1.5 T clinical scanner (General Electric, Milwaukee, WI, USA) using a self-shielding gradient set with maximum gradient of 33 mT/m and an eight-channel phased-array receive/transmit head coil. Their MRI scans were categorized according to the General and Putaminal Visual Rating Scale (Valdés Hernández et al., 2011). For this study a sample was generated containing 100 randomly selected subjects from each category of the General and Putaminal Visual Rating Scale. Two subjects were excluded due to missing MRI data and significant motion artefacts, which left 98 subjects (45 females) for the validation of the developed segmentation method.

#### Segmentation of basal ganglia T2*w hypointensities

For comparison the basal ganglia T2*w hypointensities of the subjects were segmented fully automatically and semi-automatically with the developed method, as well as manually with Analyze 10.0 (Mayo Clinic, Rochester, MN, USA) as described in Glatz et al. (2013).

The fully automated segmentation method was used to segmented basal ganglia T2*w hypointensities on the original, as well as bias-field corrected T2*w and T1w volumes with the adaptive outlier detection method since the latter yielded the most promising results *in vitro*. For the fully automated segmentation the optimal connected components filter parameters *q* were estimated with a 10-fold cross-validation method as described in Appendix C. This method optimised the connected components' filter parameters to obtain basal ganglia T2*w hypointensity masks that are most similar to those created manually.

In the semi-automated segmentation of the basal ganglia T2*w hypointensities an experienced rater (MHV) used the developed method with the adaptive version of the outlier detection method to segment basal ganglia T2*w hypointensities in all subjects on bias-field corrected T2*w and T1w volumes. For each subject the rater manually adjusted the connected components filter parameters *q*, generated the T2*w hypointensity masks, and subsequently edited the generated masks with Analyze 10.0 to add missing T2*w hypointensities or remove thresholding artefacts.

#### Numerical analysis of the volumetric and spatial mask agreement

The volumetric agreement between the fully- and semi-automatically generated masks, and manually created reference masks was quantified with the intra class correlation coefficient (ICC; agreement version), which was calculated with the R function ‘icc()’ (CRAN.R-project.org/package=psy). The spatial agreement between these masks was determined with the Jaccard index (Eq. (6)), which was also converted to the Dice coefficient as described in Shattuck et al. (2009). Furthermore, the Jaccard indices between the unfiltered basal ganglia T2*w hypointensity masks and the reference masks was quantified and related to the corresponding Jaccard indices obtained with the filtered masks to investigate the impact of the connected components filter on the spatial agreement between the generated and reference masks. The adjusted 95% confidence interval of all robust means was obtained from bootstrapping with the R function ‘boot()’ (CRAN.R-project.org/package = boot).

#### Bland–Altman analysis of the volumetric and spatial mask agreement

Modified Bland–Altman plots (Bland and Altman, 1986) were used to graphically assess the volumetric and spatial agreement between the generated and reference basal ganglia T2*w hypointensity masks. Quantile regression lines (Koenker and Hallock, 2001) were added to these plots to indicate the change of the average agreement (accuracy) and the variability of the agreement (precision) with the average mask volume, as well as to identify outliers. They were constructed by plotting the relative mask volume differences

(9) $$\Delta V_{k}^{rel}=\left(V_{T2*w,k}^{\mathrm{hypo},\mathrm{opt}}-V_{T2*w,k}^{\mathrm{hypo},ref}\right)/\bar{V_{k}}\text{,}$$

and the Jaccard indices *J*_*k*_^*opt*^ = *J*_*k*_^*opt*^(*q*^*opt*^) (Eq. (6)) over the logarithmised average mask volumes $\bar{V}=\left(V_{T2*w}^{\mathrm{hypo},\mathrm{opt}}+V_{T2*w}^{\mathrm{hypo},ref}\right)/2$, where *k* ∈ {1, 2, …, *k*^max^} represents the subject index. The normalisation of the mask volume difference with the average masks volume and the logarithmic transformation of the average mask volume mapped the mask volume differences and the average mask volumes of very large and small basal ganglia T2*w hypointensities into a similar value range. The trend of the data in the modified Bland–Altman plots was estimated with quantile regression lines (Koenker and Hallock, 2001), which were estimated for the 5th, 25th, 50th, 75th and 95th percentiles. Subjects, where neither the method nor the rater segmented basal ganglia T2*w hypointensities masks, were excluded from the plots since Eq. (9) is undefined in this case. The masks of subjects with corresponding relative volume differences or Jaccard indices outside the quantile regression lines of the 5th and 95th percentiles, i.e. the 90% range, were considered as outliers and were visually inspected.

## Results

### Validation with a custom-built phantom

#### Quality control of the CaAlg gel beads on T2*w MRI

Fig. 2B shows that the CNRs of all doped gel beads (*c* > 0 mg/l) increase approximately linearly with the contrast agent concentration since the gel beads appear increasingly hypointense with respect to the surrounding agarose on T2*w MRI. As the gel beads with a contrast agent concentration *c* = 0 mg/l appear hyperintense with respect to the surrounding agarose on T2*w MRI their CNRs are higher than those of gel beads with *c* = 1 mg/l, which appear predominantly isointense. The slopes of the robust regression lines associated with the gel bead types N/250 nm, N/1200 nm and N/120 nm + HA were 1.75, 1.34 and 0.80 l/mg, whereas their intercepts were −0.73, 0.24 and −0.67. The differences in the slopes are caused by the differences in the mass magnetic susceptibility of the contrast agents. In the case of the N/1200 nm and N/250 nm gel beads the magnetic susceptibility difference comes from the fact that the N/1200 nm particles were obtained by aggregating N/250 nm particles with additional dextran with the result that the amount of iron per N/1200 nm gel bead is slightly lower than that for the N/250 nm gel bead. Conversely, the N/1200 nm + HA gel beads have a lower magnetic susceptibility than N/1200 nm gel beads since the magnetic susceptibility of N/1200 nm and HA have opposite signs and therefore the HA crystals partly cancel the effect on the main magnetic field caused by the N/1200 nm particles.

#### Comparison of the non-adaptive and adaptive outlier detection methods

Table 3 shows that the lowest CNRs, and hence the highest T2*w thresholds relative to the mean T2*w intensity of the agarose (Eq. (3)), were obtained with the non-adaptive outlier detection method on the bias-field corrected T2*w and T1w volumes. The focal T2*w hypointensity masks created with these thresholds selected the highest number of CaAlg gel beads, however, they were also affected by the highest number of thresholding artefacts. Conversely, the lowest T2*w thresholds relative to the mean T2*w intensity of the agarose were obtained with the adaptive outlier detection method on the original T2*w and T1w volumes, which segmented the lowest number of CaAlg gel beads, as well as having the fewest thresholding artefacts. Overall, the T2*w thresholds obtained with the adaptive version of the outlier detection method on bias-field corrected T2*w and T1w volumes segmented, on average, 13% less CaAlg gel beads than the non-adaptive version, however, it also reduced the number of segmentation artefacts by, on average, 67%. In the case of the phantom this method therefore produced results that represented a trade-off in terms of the number of segmented gel beads, hence the segmentation sensitivity, and thresholding artefacts, which is also confirmed by the Jaccard indices shown in Table 3.

#### Analysis of the blooming artefacts around the CaAlg gel beads

**Fig. 3:**
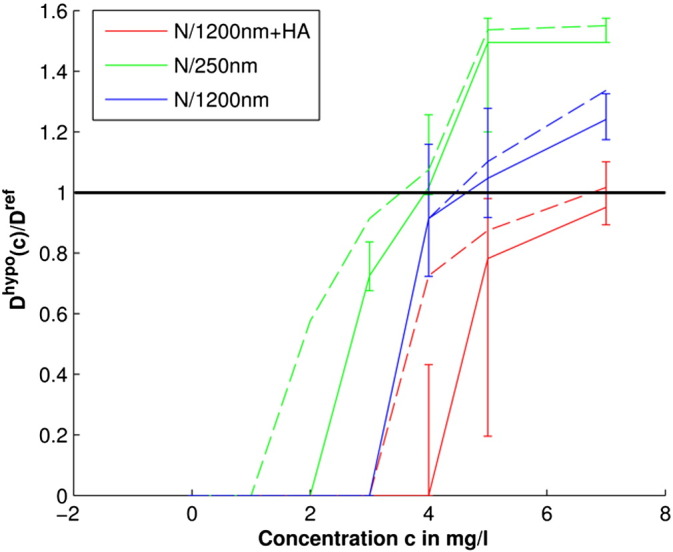
Apparent gel bead diameter increase on T2*w MRI due to blooming artefacts. The T2*w hypointensities of the doped CaAlg gel beads were segmented with the thresholds from the non-adaptive and adaptive outlier detection methods on bias-field corrected T2*w and T1w volumes. The T2*w hypointensities of the obtained masks that corresponds to gel beads were identified, their diameter measured and normalised by the average gel bead diameter *D*^*bead*^ ≈ 3 mm. This plot shows the measured diameter *D*_*h*,*l*_^*rel*^(m) over the contrast agent concentration. Dashed and solid lines refer to the results associated with the non-adaptive and adaptive outlier detection method.

Fig. 3 shows that the relative diameter of the focal T2*w hypointensities *D*_*h*,*l*_^*rel*^(*m*, *q* = 0) depends on the CaAlg gel bead type, as well as the contrast agent concentration, both factors that contribute to the volume magnetic susceptibility of the gel beads as described in Quality control of the CaAlg gel beads on T2⁎w MRI section. The apparent gel bead diameter increase on T2*w MRI can therefore be attributed to the volume of blooming artefacts around the gel beads, which are a function of the volume magnetic susceptibility (Pintaske et al., 2006). Fig. 3 also shows that the T2*w hypointensity masks of N/250 nm, N/1200 nm and N/1200 nm + HA gel beads, which were generated with T2*w thresholds from the adaptive outlier detection method, are (median [interquartile range] %) 5.4 [37.1], 10.6 [70.2], and 5.0 [5.3] % smaller than the respective masks created with T2*w thresholds from the non-adaptive outlier detection method. This approximately uniform decrease across all gel bead masks shows that the blooming artefacts cannot be selectively reduced with a subject specific threshold as was used here. The volumes of the basal ganglia T2*w hypointensity masks from the developed segmentation method are therefore influenced by the actual volume of the underlying mineralization, as well as its magnetic susceptibility.

#### Analysis of the connected components filter characteristics

**Fig. 4:**
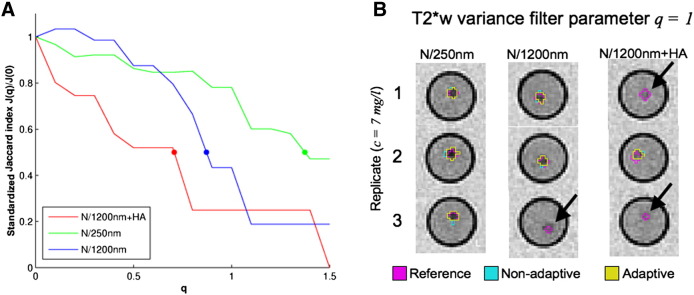
Characteristic functions of the connected components filter. The left Figure (A) shows the characteristic functions of the connected components filter for each gel bead type and the coloured dots represent the FWHM of the filter functions. These characteristic functions indicate that with increasing parameter *q* the filter predominantly rejects focal T2*w hypointensities associated with N/1200 nm and N/1200 nm + HA gel beads (black arrows) since these focal T2*w hypointensities appear more homogenous on T2*w MRI than those associated with N/250 nm gel beads. The right Figure (B) illustrates these filter properties for the case *q* = 1.

Fig. 4A shows the characteristic function of the developed connected components filter for each gel bead type. The FWHM of the characteristic filter functions associated with the N/250 nm, N/1200 nm and N/1200 nm + HA gel beads were 1.39, 0.86, and 0.72. This indicates that this filter preferentially removed T2*w hypointensities associated with N/1200 nm and N/1200 nm + HA gel beads from the T2*w hypointensity masks obtained after thresholding, which is also illustrated in Fig. 4B. The connected components filter also reduces thresholding artefacts, which can lead to maxima in the characteristic functions, as it was the case for the characteristic functions of the N/1200 gel beads. Overall these results confirm that the connected components filter preferentially retains features that appear more inhomogeneous on T2*w MRI, such as the N/250 nm gel beads, which appear as focal T2*w hypointensities with dark core regions and bright shell regions.

### Validation with subject data

#### Numerical analysis of the volumetric and spatial mask agreement

**Fig. 5:**
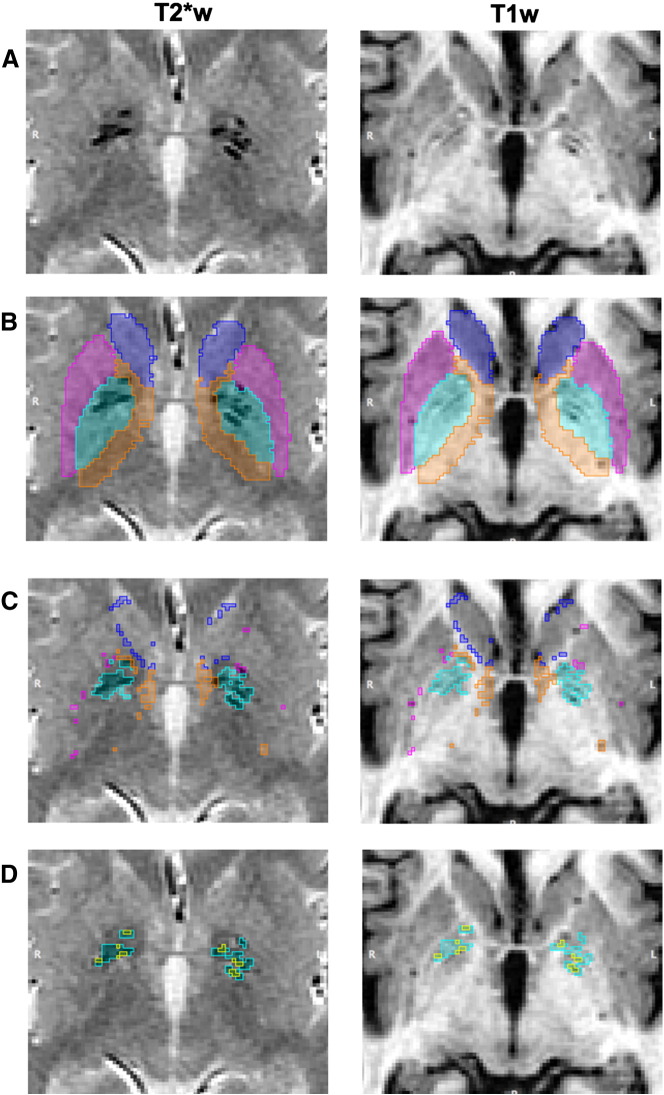
ROI, intensity distribution outliers and basal ganglia T2*w and T2*w/T1w hypointensity masks of a representative subject. The figures in the first row (A) show the basal ganglia of this subject on T2*w and T1w MRI, which are used to generate ROI masks (B). These ROI masks select most of the basal ganglia structures, as well as the adjacent internal capsule. However, the caudate and internal capsule masks appear slightly too large and also select the cerebrospinal fluid, parts of the external capsule or voxels from other adjacent structures. The T2*w and T1w intensity distribution outlier masks (C) include the basal ganglia T2*w hypointensities, which were then segmented with the derived T2*w thresholds. After reducing the thresholding artefacts with the connected components filter the final basal ganglia T2*w hypointensity mask (D, yellow and cyan masks) were obtained, which are in substantial agreement with the masks from the rater (Jaccard index = 0.93). Subregions of basal ganglia T2*w hypointensities, which appear hypointense on T1w MRI and potentially indicate advanced mineralization, are selected by the basal ganglia T2*w/T1w hypointensity masks (D, yellow masks).

The steps by which the fully and semi-automated segmentation methods create basal ganglia T2*w hypointensity masks are illustrated in Fig. 5. The results of the numerical analysis of the volumetric and spatial agreement between the manually created basal ganglia T2*w hypointensity masks and the fully and semi-automatically created masks are summarized in Table 4. This table shows that the masks created with the thresholds from the adaptive outlier detection method on the original T2*w and T1w volumes were most similar to those created manually by the experienced rater as indicated by the corresponding Jaccard indices and Dice coefficients. In the semi-automated segmentation of the basal ganglia T2*w hypointensities the rater edited a total of 20 masks which, however, lead to a minimal change in the Jaccard index compared with that produced by the fully automated segmentation using the bias-field corrected volumes. The intraclass correlation coefficient associated with the semi-automated method, on the other hand, was higher than that of the fully automated method. This indicates that manual editing of the masks by the second rater had little effect on the spatial agreement between the generated and reference masks, albeit with a marked effect on their volumetric agreement. In all cases the CNR associated with the automatically estimated thresholds (Eq. (3)), as well as the connected components' filter parameters, varied very little across the subjects. Lastly, Table 4 also shows that connected component filtering and manual editing of the unfiltered masks (*q* = 0) did not lead to a greater improvement in each subject's Jaccard index compared with connected component filtering only.

#### Bland–Altman analysis of the volumetric and spatial mask agreement

**Fig. 6:**
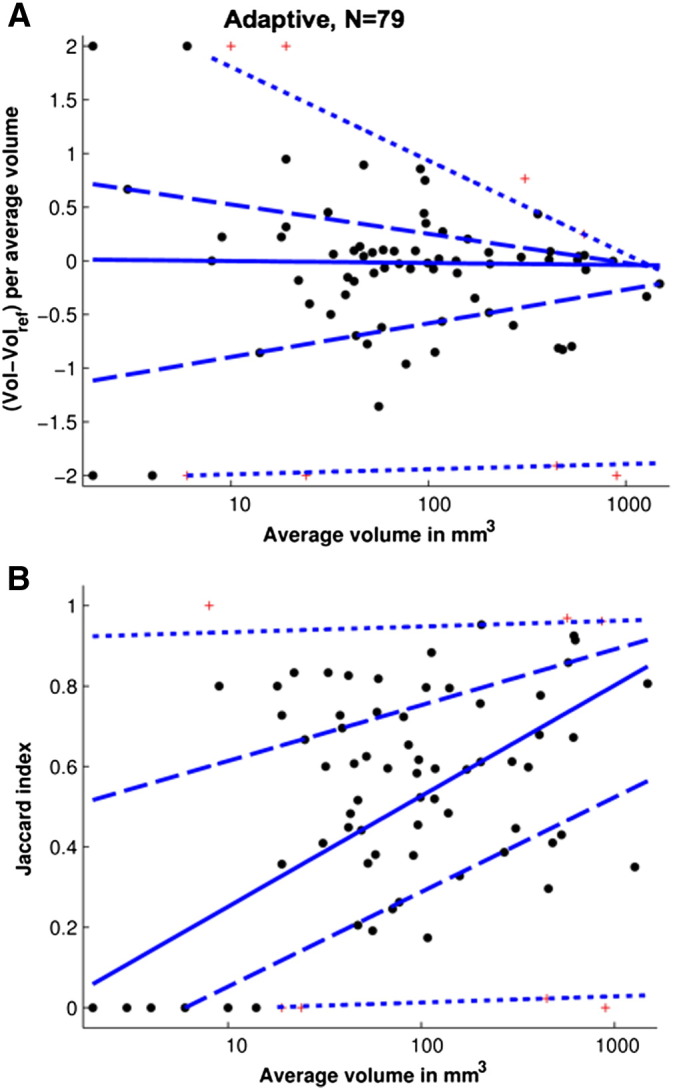
Volumetric and spatial agreement between basal ganglia T2*w hypointensity masks from the automated segmentation method and the rater. Volumetric and spatial agreement between the automatically generated basal ganglia T2*w hypointensity masks with volumes *V*_*T*2 ⁎ *w*,*k*_^*hypo*,*opt*^ and reference masks with volumes *V*_*T*2 ⁎ *w*,*k*_^*hypo*,*ref*^ from the rater were assessed with modified Bland–Altman plots. The upper plots (A) show the mask volume differences over the average mask volumes and the lower plot (B) the Jaccard indices (Eq. (6)) over the logarithmised average mask volumes. Subjects for which neither the rater nor the method generated basal ganglia T2*w hypointensity masks were excluded since the y-axis values were mathematically not defined. The trend in the data was estimated with quantile regression lines (Koenker and Hallock, 2001), which were calculated for the 50th percentile (solid line), 25th and 75th percentiles (dashed lines), and 5th and 95th percentiles (dotted lines). Data points outside the 90% range were considered outliers and are shown as red crosses. Quantile regression lines were censored if their y-axis values exceeded the valid y-axis value ranges.

The volumetric and spatial agreement between the basal ganglia T2*w hypointensity masks, which were automatically created on the bias-field corrected T2*w and T1w volumes, and the corresponding reference masks was assessed with the Bland–Altman plots in Fig. 6. The plot in Fig. 6A indicates that the average mask volume difference, as indicated by the median regression line, is close to zero and slightly decreases with the average mask volume. This indicates that the generated and reference mask volumes were, on average, very similar. However, the masks for smaller and larger basal ganglia T2*w hypointensities were slightly too large and slightly too small. Conversely, Fig. 6B shows that there was largely no spatial agreement between the generated and reference masks for very small basal ganglia T2*w hypointensities. However, the spatial agreement between generated and reference masks increased markedly with the average mask volume to Jaccard indices above 0.7, corresponding to an 80% overlap of these masks. Furthermore, both figures show that the variability of the volumetric and spatial agreement decreased with the average mask volume. Overall, these results indicate that for subjects with large basal ganglia T2*w hypointensities there was substantial volumetric and spatial agreement between the automatically generated and the reference masks from the experienced rater.

Data points outside the 5th and 95th percentile regression lines in Fig. 6A and data points below the 5th percentile regression line in Fig. 6B represent subjects for which the method failed to produce masks. Visual inspection of the generated and reference masks of these subjects found that most of the T2*w hypointensities were very small, appeared isointense on T1w MRI, and were associated with a low CNR and fuzzy boundaries on T2*w MRI. In the case of one subject the T2*w MRI was heavily compromised by susceptibility artefacts, and in other two cases the T2*w and T1w intensity distributions of the normal-appearing basal ganglia tissue were either bimodal or did not resemble normal distributions. The latter violated the main assumptions of the outlier detection methods and therefore there was a marked difference between the automatically estimated T2*w thresholds and those produced by the rater.

## Discussion

In this study we developed and validated a novel method for the automatic segmentation of multifocal T2*w hypointensities in the basal ganglia and adjacent internal capsule which are believed to arise from mineral deposits in and around the penetrating arterioles and perivascular spaces. This method uses an adaptive outlier detection method to derive T2*w and T1w thresholds from bivariate T2*w and T1w intensity distributions of individual basal ganglia structures. The CNRs associated with these thresholds (Eq. (3)) are insensitive to a change in the mean T2*w and T1w intensity of a structure, as it is the case in ageing due to iron accumulation. These thresholds are then used to segment all focal T2*w hypointensities of a structure and a connected components filter subsequently removes focal T2*w hypointensities with standardised T2*w intensity variances below automatically derived thresholds to reduce thresholding artefacts. The output of this method are basal ganglia T2*w and T2*w/T1w hypointensity masks, where the latter presumably indicates regions of advanced mineralisation. The effects of the outlier detection method and the connected components filter on the segmentation results were investigated with a custom-built phantom containing different models of basal ganglia mineral deposits. The method was also validated with MRI data from 98 community-dwelling older subjects in their seventies to assess its ability to generate similar masks to those produced by an experienced rater.

The phantom experiments show that blooming artefacts around the CaAlg gel beads increase their apparent diameter on T2*w MRI by up to 1.6 times. The volume of the generated basal ganglia T2*w hypointensity masks therefore reflects not only the volume of the underlying mineral deposits but also their magnetic susceptibility (Bos et al., 2003; Pintaske et al., 2006). In previous studies (Penke et al., 2012), the magnetic susceptibility therefore likely acted as an additional weight in the correlation analysis. Hence mineral deposits with the same volume, but different magnetic susceptibility, possibly have a different impact on the correlation result. As the magnetic susceptibility of mineral deposits can be determined with gradient-echo MRI it should be possible to develop a correction method for blooming artefacts (McAuley et al., 2011). Such a method could help to clarify the specific impact of the chemical composition and extent of mineral deposits in the ageing brain.

The unsupervised multivariate outlier detection methods rely on the assumption that the co-registered T2*w and T1w signal intensities of normal-appearing tissue resemble bivariate normal distributions with robust distances which in turn resemble non-central χ^2^ distributions (Hardin and Rocke, 2005). Although this assumption was not explicitly checked, it can be concluded that it was reasonably well satisfied since the automated method produced segmentation results that were in substantial agreement with the reference masks of the phantom and subjects. Furthermore, the T2*w sequence used in this study (GRASS) was chosen over spoiled gradient echo sequences since it can produce T2*w images with comparatively higher SNR (Bernstein et al., 2004), which helps in assuring that the T2*w intensities of normal-appearing tissue are normally distributed (Gudbjartsson and Patz, 1995). Additionally, the non-anatomical intensity variations caused by the B1 field inhomogeneities (bias field) were corrected on T2*w and T1w MRI, which can also significantly distort the image intensities, and hence reduce the quality of the segmentation results as demonstrated with the phantom results. Recent performance studies also show that the adaptive variant of the unsupervised outlier detection method produces better results than the non-adaptive variant in the case of heavy tailed or skewed multivariate distributions (Filzmoser, 2005). However, further studies are required to explore the impact on the segmentation results if the T2*w and T1w signal intensity distributions deviate from bivariate normal distributions and if other multivariate outlier detection methods, such as non-parametric methods (Ben-Gal, 2010), could improve the segmentation results.

The developed automated segmentation method for multifocal T2*w hypointensities employs a novel filter, which preferentially removes connected components that appear more homogenous on T2*w MRI, i.e. are associated with a standardised T2*w intensity variance below a threshold q. The subject results confirm that this filter markedly improved the spatial agreement between the automatically generated and reference masks. These findings therefore suggest that the rater, after segmenting basal ganglia T2*w hypointensities with the semi-automated thresholding method (Valdés Hernández et al., 2011), also excluded individual T2*w hypointensities that appeared more homogenous on T2*w MRI. Additionally, the phantom experiments showed that the filter preferentially removed T2*w hypointensities associated with gel beads that were doped with N/1200 nm, and especially with N/1200 nm and HA microcrystals. This suggests that the filter possibly excludes mineral deposits with a specific chemical composition, such as mineral deposits with calcification, since this makes them appear more homogenous on T2*w MRI. However, further work is required to explain the effects of the chemical composition of trace metal deposits on the T2*w intensity variance. So far, the local intensity variance has only been used as a quality measure for T1w MRI (Aja-Fernández et al., 2006).

In this study the optimal connected components filter parameter for segmenting the basal ganglia T2*w hypointensities in the LBC1936 subjects was derived in a 10-fold cross validation with the reference masks of an experienced rater. However, as the optimal parameter depends on the outlier detecting method and potentially other factors, such as the T2*w sequence and main magnetic field strength, it might be different for subsequent studies. The optimal connected components filter parameter can then be estimated in two ways. As in this study, a representative and random subject subset can be constructed, where an experienced rater segments the basal ganglia T2*w hypointensities and the optimal filter parameter is estimated with cross-validation. If masks from an experienced rater are not available, a heuristic method could possibly be used to estimate the optimal connected components parameter. A phantom with the same basal ganglia mineralization models as employed in the current study is needed to derive the characteristic connected components filter functions (Fig. 4A). Here an optimal value of the filter parameter was identified as where the difference between the characteristic filter function of the gel beads with N/250 nm and the average filter function of the gel beads with N/1200 nm and N/1200 nm + HA was maximal. The additional advantage of this approach is that the detection sensitivity for focal mineralizations, as well as the size of the blooming artefacts can also be quantified.

This study has several strengths. Firstly, our method was validated with standard structural T2*w and T1w volumes from both a custom-designed phantom and from a random sample of community-dwelling older subjects with a very narrow age range. Secondly, the subject sample was carefully chosen to include a wide range of basal ganglia T2*w hypointensities based on their appearance and morphology. Finally, the experienced rater who manually segmented these basal ganglia T2*w hypointensities was not involved in the selection process to ensure an unbiased reference. This study design therefore ensures that the developed automated segmentation method for T2*w hypointensities is largely unbiased and can therefore be readily evaluated in further studies of old age.

The presented automated method also has several limitations. Firstly, it requires co-registered T1w volumes to (i) automatically generate the ROI masks for all basal ganglia structures with FSL FIRST (Patenaude et al., 2011), (ii) exclude artefacts, such as chronic haemorrhages, and (iii) segment subregions of basal ganglia T2*w hypointensities which might represent regions of advanced mineralization. High-resolution T1w volumes are typically part of clinical MRI protocols and a sufficiently accurate registration to corresponding T2*w volumes can often be achieved if the acquisition parameters are optimised. However, in situations where T1w volumes are missing or major registration artefacts are present the developed segmentation method cannot be used in its present form. Secondly, the volume of basal ganglia T2*w hypointensities, and hence the volume of the generated masks, is affected by the blooming artefact (Bos et al., 2003; Pintaske et al., 2006). As noted above, the blooming artefact depends on the magnetic susceptibility of the underlying tissue. However, it also depends on scanner and MRI sequence parameters (Pintaske et al., 2006), such as the main magnetic field strength B0, the orientation of the plane of view relative to B0, the echo time and the voxel size. Studies which acquire MRI data on different scanners, with different T2*w sequences or T2*w sequence parameters, therefore have to correct their results for these factors. Finally, the MCD method (Rousseeuw, 1999), which is part of the unsupervised outer detection method for estimating the location and scatter of the bivariate T2*w and T1w distributions, enforces an upper size limit on the basal ganglia T2*w hypointensities, since it can only tolerate up to 50% outliers. Therefore this method misclassifies basal ganglia T2*w hypointensities that are larger than half the volume of the surrounding normal-appearing tissue, as was the case for one subject in this study with very large bilateral basal ganglia T2*w hypointensities. However, very large basal ganglia T2*w hypointensities possibly have a non-ischemic aetiology (Janaway et al., 2014; Morris et al., 1992) and therefore should be analysed separately.

In conclusion, this paper presents a novel automated method for segmenting basal ganglia T2*w hypointensities which consists of an unsupervised outlier detection method and a connected components filter to reduce thresholding artefacts. Data from a custom-built MRI phantom with mineral deposit models and a random sample of older subjects from the LBC1936 showed that this method was able to generate basal ganglia T2*w hypointensity masks that were in substantial agreement with manually created reference masks from an experienced rater. This method could therefore be potentially useful in future studies investigating relationships between basal ganglia T2*w hypointensities and other features of small vessel disease and the ageing brain. However, further testing of this method in other independent data sets is still required to confirm its general validity.

## Figures and Tables

**Fig. 7:**
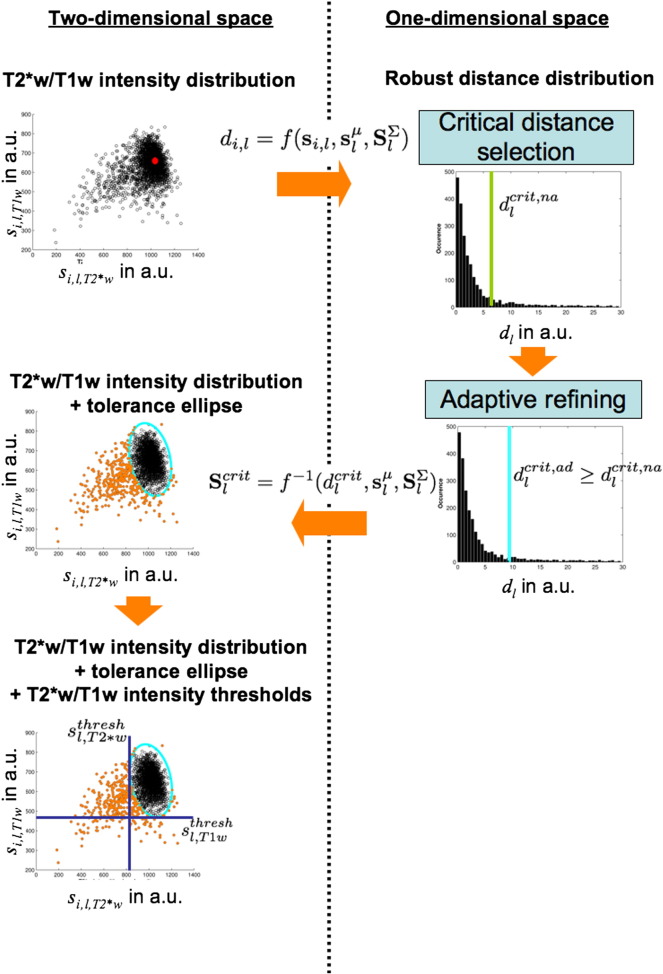
Automatic T2*w and T1w intensity threshold selection based on the T2*w/T1w intensity distribution outliers of a ROI. The T2*w/T1w intensity distribution of a ROI is mapped from the two-dimensional intensity distribution space to the one-dimensional robust distance space by calculating the robust distance (Hardin and Rocke, 2005) between each T2*w/T1w intensity pair and the centre of the distribution (red dot). An initial critical robust distance *d*_*l*_^*crit*^ is then estimated from a χ^2^ distribution (degrees of freedom = 2). In the case of the adaptive variant of the outlier detection method this distance is further refined (Filzmoser et al., 2005). The final critical distance is mapped back to the two-dimensional intensity distribution space where it represents a tolerance ellipse (cyan). T2*w/T1w intensity tuples on and outside this tolerance ellipse (orange dots) are considered to be associated with focal features, such as basal ganglia T2*w hypointensities, whereas those inside the ellipse are considered to be associated with normal-appearing tissue. The hypointense outlier intensities on T2*w MRI and T1w MRI are subsequently extracted with T2*w and T1w thresholds (purple) which are derived from the tolerance ellipse.

**Fig. 8:**
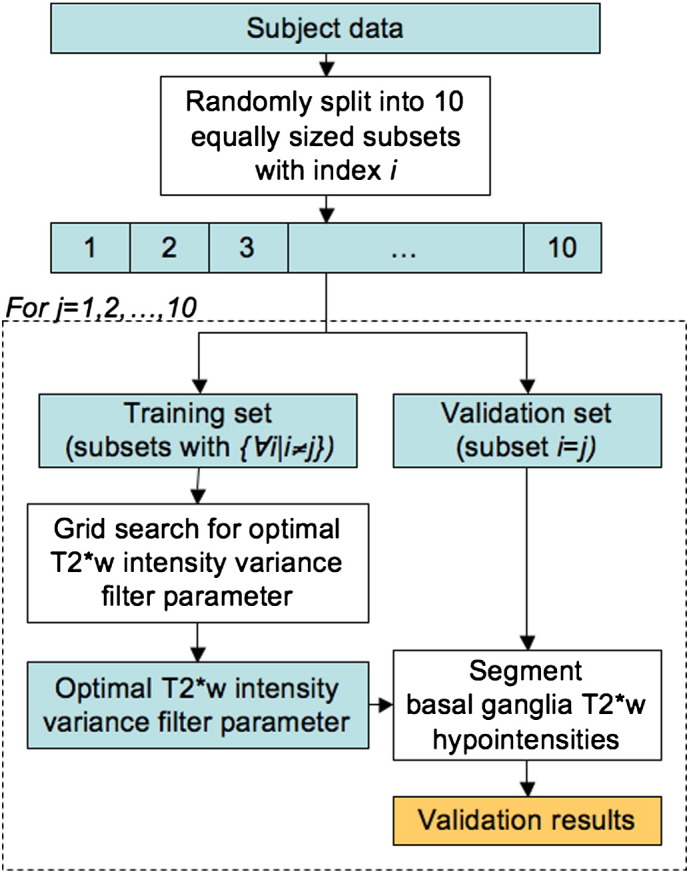
Optimal selection of the connected component filter parameter q with 10-fold cross-validation. This flow diagram illustrates how the subject data was split into training and validation sets in the 10-fold cross-validation. The optimal connected component filter parameter *q*^*opt*^ was then estimated in a grid search for each training set. This parameter represents the connected component filter parameter *q* that maximises the Jaccard index *J*^*med*^(*q*) (Eq. (C.1)), and hence maximises the spatial agreement between the automatically generated masks for the training set subjects and the corresponding reference masks from the rater. The estimated parameters *q*^*opt*^ were subsequently used to segment the basal ganglia T2*w hypointensities of the subjects in the corresponding validation sets.

**Table 1:** Composition of the sodium alginate solutions for creating the calcium alginate gel beads used as mineral deposit models. A 2% (w/v) sodium alginate solution was used as the base solution for creating all gel beads (Xie et al., 2010). The MRI contrast agents Nanomag 250 and 1200 nm, as well as the hydroxyapatite precursor, were added in the following concentrations to the base solution to create gel beads containing iron and hydroxyapatite nanocrystals, which were then used as mineral deposit models.

Gel bead type	Sodium alginate solution additives	
	MRI contrast agent	Hydroxyapatite precursor
N/250 nm	0, 1, 2, ..., 7 mg/l Nanomag 250 nm	None
N/1200 nm	0, 1, 2, ..., 7 mg/l Nanomag 1200 nm	None
N/1200 nm + HA	0, 1, 2, ..., 7 mg/l Nanomag 1200 nm	200 mmol/l Na_2_HPO_4_

**Table 2:** MRI sequence parameters for scanning the phantom and LBC1936 subjects. The complete protocol for LBC1936 subjects is described in Wardlaw et al. (2011).

Sequence	IR-prep FGRE (3D)	GRASS (2D)
Contrast type	T1-weighted (T1w)	T2*-weighted (T2*w)
Flip angle in degrees	8	20
TI/TR in ms	500/9.8	–/940
Bandwidth in Hz/pixel	122	98
TE in ms	4	15

*MRI phantom*^a^		
FOV in mm^2^	192 × 192	192 × 192
Orientation	Axial	Axial
Slice thickness in mm	1.2	2.4
Acquisition matrix (effective and final)	256 × 256	256 × 256

*Subjects*		
FOV in mm^2^	256 × 256	256 × 256
Orientation	Coronal	Axial
Slice thickness in mm	1.3	2
Effective acquisition matrix	192 × 192	256 × 192
Final acquisition matrix^b^	256 × 256	256 × 256

a The T1w and T2*w sequences were configured to image the same phantom volume.

b After interpolation by the scanner software.

**Table 3:** Comparison of the outlier detection methods. The focal T2*w hypointensities of the phantom were segmented on the original and bias-field corrected T2*w and T1w volumes with the T2*w thresholds from the non-adaptive and adaptive version of the outlier detection method. This table shows the minimum CNR of the segmented gel beads and the CNRs of the T2*w thresholds (Eq. (3)), as well as the average Jaccard index, which quantifies the spatial agreement between the generated and reference masks of each ROI. Furthermore it includes the average number of segmented focal T2*w hypointensities per ROI that was associated with gel beads (total number of gel beads per ROI = 7) and thresholding artefacts.

Outlier detection method	Non-adaptive		Adaptive	
Bias-field correction	None	N4	None	N4
Minimum CNR of CaAlg gel beads	5.60 ± 0.80	4.58 ± 0.42	7.39 ± 1.45	5.19 ± 0.55
CNR of T2*w threshold	5.21 ± 0.40	4.51 ± 0.38	6.47 ± 0.87	5.02 ± 0.44
Jaccard index	0.16 ± 0.08	0.21 ± 0.07	0.12 ± 0.10	0.22 ± 0.09
Segmented gel beads	1.33 ± 0.87	2.56 ± 1.24	0.89 ± 0.93	2.22 ± 1.20
Thresholding artefacts	4.67 ± 2.96	20.44 ± 4.90	2.33 ± 1.41	6.67 ± 3.54

**Table 4:** Basal ganglia T2*w hypointensity segmentation statistics. Basal ganglia T2*w hypointensities were segmented with the fully- and semi-automated methods. In the former case the features were segmented on the original and bias-field corrected T2*w and T1w volumes, whereas in the latter case they were segmented on the bias-field corrected T2*w and T1w volumes. The T2*w and T1w thresholds were estimated with the adaptive outlier detection method and the connected components filter parameter *q* was either chosen with the 10-fold cross-validation method or manually by the rater. In the semi-automatic segmentation method, the rater also edited the generated masks to add basal ganglia T2*w hypointensities that were missed by the method and to remove thresholding artefacts. This table shows the robust mean, standard deviation (SD) and 95% confidence interval (CI) of the robust mean of the filter parameter *q*, the CNR associated with the T2*w threshold (Eq. (3)), as well as measures that quantify the volumetric and spatial agreement between the generated and reference masks, which were manually created by a second experienced rater.

Method	Fully-automated				Semi-automated	
Bias-field correction	None		N4		N4	
Statistics	Mean ± SD	95% CI	Mean ± SD	95% CI	Mean ± SD	95% CI
Parameter *q*	0.90 ± 0.00	N/A	0.80 ± 0.00	N/A	0.80 ± 0.00	N/A
CNR	8.8 ± 1.3	8.6, 9.1	7.8 ± 1.4	7.7, 8.1	7.8 ± 1.4^b^	7.7, 8.1^b^
Jaccard	0.67 ± 0.40	0.51, 0.75	0.62 ± 0.40	0.53, 0.74	0.62 ± 0.41	0.48,0.70
Δ Jaccard^a^	0.03 ± 0.34	0, 0.06	0.03 ± 0.23	0, 0.07	0.03 ± 0.30	0,0.05
Dice	0.80 ± 0.29	0.69, 0.83	0.77 ± 0.30	0.69, 0.86	0.77 ± 0.33	0.63,0.83
ICC	0.69	0.24, 0.91	0.70	0.23, 0.93	0.74	0.54,0.90

a Relative to the corresponding Jaccard indices obtained at *q* = 0.

b Same values as obtained with the automated method on bias-field corrected volumes.

## Appendix A Unsupervised outlier detection method for bivariate data

The unsupervised outlier detection method (Filzmoser et al., 2005) that was applied in this study was originally developed for exploration geochemistry. Fig. 7 shows an overview of this method that in this study was adapted for MRI data, where the input are co-registered T2*w/T1w intensities from a ROI. The method involves two steps. First it estimates the tolerance ellipse from the T2*w/T1w intensity distribution for classifying these T2*w/T1w intensities into outlier and normal-appearing tissue intensities. Secondly, T2*w/T1w thresholds are derived from the obtained tolerance ellipse for the segmentation of T2*w hypointensities and their subregions that appear hypointense on T1w MRI.

### A.1 Estimation of the tolerance ellipse from the T2*w/T1w intensity distribution

As shown in Fig. 7 the bivariate T2*w/T1w intensity distribution are first transformed into a univariate robust distance distribution from which a critical robust distance is derived, which represents the tolerance ellipse in the bivariate intensity distribution space.

#### A.1.1 Transformation into the univariate robust distance space

Given the T2*w/T1w intensity tuples of all voxels *i* ∈ *M*

(A.1) $$s_{i}=\left(\begin{array}{c} s_{i,T2*w}\\ s_{i,T1w} \end{array}\right)\text{,}$$

the mean of all ROI tuples *i* ∈ *M*_*l*_^ROI^ is denoted by

(A.2) $$s_{l}^{\mu }=\left(\begin{array}{c} s_{l,T2*w}^{\mu }\\ s_{l,T1w}^{\mu } \end{array}\right)\text{,}$$

and their covariance is denoted by

(A.3) $$S_{l}^{\Sigma }=\left(\begin{array}{cc} {\left(s_{l,T2*w}^{\sigma }\right)}^{2} & r_{l}^{\rho }s_{l,T2*w}^{\sigma }s_{l,T1w}^{\sigma }\\ r_{l}^{\rho }s_{l,T2*w}^{\sigma }s_{l,T1w}^{\sigma } & {\left(s_{l,T1w}^{\sigma }\right)}^{2} \end{array}\right)\text{,}$$

where *s*_*l*,T2 ⁎ w_^*σ*^, *s*_*l*,T1w_^*σ*^ are the respective standard deviations and *r*_*l*_^*ρ*^ is the cross correlation coefficient. In this study, the mean and covariance were estimated with the Minimum Covariance Determinant (MCD) estimator (Rousseeuw, 1999), which provides robust estimates of these parameters and resists up to 50% outliers. The robust mean and covariance were then used to calculate the robust distances (Rousseeuw and Van Zomeren, 1990)

(A.4) $$d_{i}\left(s_{i}\right)={\left(s_{i}-s_{l}^{\mu }\right)}^{T}{\left(S_{l}^{\Sigma }\right)}^{-1}\left(s_{i}-s_{l}^{\mu }\right)\text{,}$$

which represent the distances between the T2*w/T1w intensity distribution centre and each T2*w/T1w intensity tuple *i* ∈ *M*_*l*_^ROI^.

#### A.1.2 Estimating a critical robust distance from the robust distance distribution

The unsupervised outlier detection method is based on the assumption that the T2*w/T1w intensities follow a bivariate normal distribution, which is sufficiently satisfied in ROIs with signal-to-noise ratios (SNRs) larger than 2 (Gudbjartsson and Patz, 1995). The robust distances (Eq. (A.4)) then follow a non-central chi-square distribution *χ*^2^(*p*, *df* = 2) with two degrees of freedom (Hardin and Rocke, 2005). Signal intensity outliers have the highest robust distances and hence are found in the right tail of the *χ*^2^ distribution above a critical distance

(A.5) $$d_{l}^{\mathrm{crit},na}=\chi^{2}\left(p=0.975,df=2\right)\text{.}$$

The selection of the critical distance is crucial for separating extreme values of distributions and outliers, while Filzmoser (2005) showed that a refinement of the critical distance with an adaptive method is more robust and leads to better results. This adaptive outlier detection method uses the critical distance of the non-adaptive method *d*_*l*_^*crit*,*na*^ as an initial estimate, which is then refined by considering the sample size *n*_*l*_ of the T2*w/T1w intensity distribution. Firstly, the difference in the tails of the cumulative distribution function *G* of a chi-square distribution *χ*^2^(*df* = 2) and the empirical distribution $G_{n_{l}}$ of the robust distances *d*_*i*_ of ROI *l* is calculated. If no outliers are present then this difference denoted by

(A.6) $$\Delta G_{n_{l}}^{\mathrm{tail}}=\underset{d\geq d_{l}^{\mathrm{crit},na}}{\,\sup}{\left(G\left(d\right)-G_{n_{l}}\left(d\right)\right)}^{+}\text{,}$$

is expected to be lower than a critical difference $\Delta G_{n_{l}}^{\mathrm{tail},\mathrm{crit}}$. Filzmoser et al. (2005) derived the relationship between the sample size and the critical difference $\Delta G_{n_{l}}^{\mathrm{tail},\mathrm{crit}}$ for normal distributions in computer simulations. For bivariate normal distributions this relationship is

(A.7) $$\Delta G_{n_{l}}^{\mathrm{tail},\mathrm{crit}}=\frac{0.234}{\sqrt{n_{l}}}\text{.}$$

The refined critical robust distance is then

(A.8) $$d_{l}^{\mathrm{crit},\mathrm{ad}}={G_{n}}^{-1}\left(1-\alpha_{n_{l}}^{\mathrm{tail}}\right)\;\mathrm{with}\;\alpha_{n_{l}}^{\mathrm{tail}}=\left\{\begin{array}{cc} 0 & \Delta G_{n_{l}}^{\mathrm{tail}}\leq \Delta G_{n_{l}}^{\mathrm{tail},\mathrm{crit}}\\ \Delta G_{n_{l}}^{\mathrm{tail}} & \Delta G_{n_{l}}^{\mathrm{tail}}>\Delta G_{n_{l}}^{\mathrm{tail},\mathrm{crit}} \end{array}\right)\text{,}$$

which has the desired property that for *n*_*l*_ → ∞ the critical robust distance *d*_*l*_^*crit*,*ad*^ is equal to the robust distance of the sample that is furthers away from the distribution centre since a distribution with infinite number of sample does not have outliers but only extreme values.

### A.2 Deriving T2*w and T1w thresholds from the critical robust distance

As illustrated in Fig. 7, the T2*w/T1w thresholds represent the minimum of all points of the estimated tolerance ellipse of the T2*w/T1w intensity distribution. Given the previously estimated critical robust distance *d*_*l*_^*crit*,*m*^ with *m* ∈ {*na*, *ad*}, these thresholds can be calculated by solving Eq. (A.4) for **s**_*i*_ and calculating the minimum for each dimension

(A.9) $$\begin{array}{l} s_{l,T2*w}^{\mathrm{thresh},m}=s_{l,T2*w}^{\mu }-s_{l,T2*w}^{\sigma }{\overset{⌣}{d}}_{l}^{\mathrm{crit},m}\\ s_{l,T1w}^{\mathrm{thresh},m}=s_{l,T1w}^{\mu }-s_{l,T1w}^{\sigma }{\overset{⌣}{d}}_{l}^{\mathrm{crit},m}\text{,} \end{array}$$

where ${\overset{⌣}{d}}_{l}^{\mathrm{crit},m}=\sqrt{d_{l}^{\mathrm{crit},m}}$ represents the z-score associated with the critical robust distance.

## Appendix B Segmentation of basal ganglia T2*w hypointensities

Initial basal ganglia T2*w hypointensity masks were generated by thresholding the T2*w intensities of all ROIs with the lowest T2*w threshold, i.e. that of the globus pallidus. The connected components of these masks were then identified and filtered based on their T2*w intensity variance, which reduces segmentation artefacts, mostly due to image noise. Subregions of the final masks that appear hypointense on T1w MRI were also segmented since they possibly indicate advanced mineralization, such as calcification (Valdés Hernandéz et al., 2014).

Initial basal ganglia T2*w hypointensity masks

(B.1) $$M_{T2*w}^{\mathrm{hypo},\mathrm{init}}=\left\{i\in M^{ROI}|s_{i,T2*w}<s_{gp,T2*w}^{\mathrm{thresh},m}\right\}\text{,}$$

were obtained with the globus pallidus T2*w threshold *s*_*gp*,T2 ⁎ w_^*thresh*,*m*^ (Eq. (A.9)). Then the connected components (six-connected neighbourhood) of these masks, with the unique labels *h* ∈ {1, 2, …, *h*^max^} and the masks $M_{T2*w}^{\mathrm{hypo},\mathrm{init}}\subset M_{T2*w}^{\mathrm{hypo},\mathrm{init}}$, were identified with the Matlab function ‘bwlabeln()’. The locations of these connected components within the basal ganglia were determined with the ROI mask set *M*^ROI^ as described in Glatz et al. (2013). This yielded sets *H*_*l*_^*init*^ that contained the indices of all connected components located within each ROI *l*.

The standardised T2*w intensity variance of a connected component was defined as

(B.2) $$q_{l,h}=\frac{{\left(s_{h,T2*w}^{\mathrm{hypo},\sigma }\right)}^{2}}{{\left(s_{l,T2*w}^{\mathrm{norm},\sigma^{loc}}\right)}^{2}}\;\mathrm{with}\;h\in H_{l}^{\mathrm{init}}\text{.}$$

The nominator represents the T2*w intensity variance of a connected component and the denominator represents the local T2*w intensity variance of the surrounding normal-appearing tissue. The latter was defined as

(B.3) $$s_{l,T2*w}^{\mathrm{norm},\sigma^{loc}}={\left(\left\{s_{i,T2*w}^{\sigma^{loc}}|i\in M_{l}^{\mathrm{norm}}\right\}\right)}_{0.5}\text{,}$$

where the local T2*w intensity variances of each voxel $s_{i,T2*w}^{\mathrm{norm},\sigma^{loc}}$ were obtained with the Matlab function ‘stdfilt()’ and the normal-appearing tissue mask

(B.4) $$M_{l}^{\mathrm{norm}}=\left\{i\in M_{l}^{ROI}|d_{i}<d_{l}^{\mathrm{crit},m}\right\}\text{.}$$

The standardised T2*w intensity variance *q*_*l*,*h*_ therefore represents a measure of how inhomogeneous a connected component appears on T2*w MRI relative to the surrounding normal-appearing tissue.

To reduce segmentation artefacts the connected component filter of the method removed connected components of the initial basal ganglia T2*w hypointensity masks that appeared too homogenous on T2*w MRI, i.e. connected components with a standardised T2*w intensity variance below a threshold *q*. This yielded the final basal ganglia T2*w hypointensity masks for each ROI *l*

(B.5) $$M_{l,T2*w}^{\mathrm{hypo}}=\underset{h\in H_{l}}{\cup }M_{h,T2*w}^{\mathrm{hypo},\mathrm{init}}\;\mathrm{with}\;H_{l}=\left\{h\in H_{l}^{\mathrm{init}}|q_{l,h}>=q\right\}\text{.}$$

Lastly, basal ganglia T2*w/T1w hypointensity masks

(B.6) $$M_{T1w}^{\mathrm{hypo}}=\underset{\forall l}{\cup }\left\{i\in M_{l,T2*w}^{\mathrm{hypo}}|s_{i,T1w}<s_{l,T1w}^{\mathrm{thresh},m}\right\}\text{,}$$

which select subregions of the final basal ganglia T2*w hypointensity masks that appear hypointense on T1w MRI, were segmented by thresholding the T1w intensities selected by *M*_*l*,*T*2 ⁎ *w*_^*hypo*^ with the respective thresholds *s*_*l*,*T*1*w*_^*thresh*,*m*^ (Eq. (A.9)).

## Appendix C Ten fold cross-validation method for estimating the optimal connected components filter parameter q

Fig. 8 illustrates the method for estimating the optimal connected component filter parameter *q*^*opt*^ for a given subject sample. Firstly, the subject sample was randomized and split into 10 approximately equally sized subsamples. Each subsample was used as a validation set, whereas the remaining nine subsamples were combined into a training set. The optimal filter parameter of a given training set was then estimated with a grid search (Bergstra and Bengio, 2012), where masks *M*_T2 ⁎ w,*k*_^*hypo*^(*b*, *m*, *q*) for the basal ganglia T2*w hypointensities of the training set subjects with index *k* were generated with the connected components filter parameters *q* = 0, 0.1, …, 1.5. The average spatial agreement between the generated and corresponding reference masks was quantified with the median Jaccard index

(C.1) $$J^{med}\left(q\right)={\left(J_{k}\left(q\right)\right)}_{0.5}\;\mathrm{with}\;J_{k}\left(q\right)=\frac{\left|M_{T2*w,k}^{\mathrm{hypo}}\left(b,m,q\right)\cap M_{T2*w,k}^{\mathrm{hypo},ref}\right|}{\left|M_{E2*w,k}^{\mathrm{hypo}}\left(b,m,q\right)\cup M_{T2*w,k}^{\mathrm{hypo},ref}\right|}\text{,}$$

where *M*_T2 ⁎ w,*k*_^*hypo*,*ref*^ denotes the manually created reference masks. The filter parameter that was associated with the highest median Jaccard index

(C.2) $$q^{\mathrm{opt}}=\underset{\forall q}{\,\arg\,\max}J^{med}\left(q\right)\text{,}$$

was selected as the optimal filter parameter for the corresponding validation set and subsequently used to create the basal ganglia T2*w hypointensity masks *M*_T2 ⁎ w,*k*_^*hypo*,*opt*^ = *M*_T2 ⁎ w,*k*_^*hypo*^(*b*, *m*, *q* = *q*^*opt*^) of the corresponding validation set subjects, as well as the T2*w/T1w hypointensity masks *M*_T1w,*k*_^*hypo*,*opt*^ = *M*_T1w,*k*_^*hypo*^(*b*, *m*, *q* = *q*^*opt*^).
